# Plant organ and tip growth

**DOI:** 10.1093/jxb/eraa163

**Published:** 2020-04-23

**Authors:** Kris Vissenberg, Nathalie Gonzalez

**Affiliations:** 1 Integrated Molecular Plant Physiology Research, Department of Biology, University of Antwerp, Antwerp, Belgium; 2 Plant Biochemistry and Biotechnology Lab, Department of Agriculture, Hellenic Mediterranean University, Heraklion, Crete, Greece; 3 INRAE, Université de Bordeaux, BFP, Villenave d’Ornon, France

**Keywords:** Cell expansion, cell proliferation, development, environmental cues, growth, growth regulation, hormones, leaf, photosynthesis, pollen, root, root hairs, self-incompatibility, tip growth

**Plant growth is a dynamic and multifactorial trait that is highly regulated by both internal and external cues. Amongst other things, these influence gene expression, protein abundance, post-translational modifications, cell wall composition, and ion dynamics, all of which affect the extent of growth. To obtain a comprehensive view of the complex regulatory mechanisms that control growth, complementary approaches that focus on different scales are needed. This special issue comprises eight articles that present recent advances in our knowledge of the regulation of plant organ and tip growth.**

The multifaceted process of growth involves a complex and interconnected network of regulators that integrate a variety of signals to drive and/or adapt growth. Plants grow mainly post-embryonically, forming two types of organs: those such as leaves with a determinate growth and a fixed final size, and those such as roots with an indeterminate growth and thus a theoretical unlimited growth potential. In contrast to most plant cells where growth occurs across all or most surfaces of the cell, root hairs and pollen tubes grow by tip growth. This highly polarized secretion of new cell wall material at the tip allows targeted growth towards water and nutrients in case of root hairs, and towards egg cells in the case of pollen tubes. This special issue presents recent advances in the study of these different organ types and discusses new insights regarding their growth regulatory mechanisms. The papers are from the *JXB*-sponsored ‘Plant Organ Growth Symposium’ and ‘Tip growth in plant biology’ sessions that were held in Bordeaux (April 2019) and during the Society of Experimental Biology (SEB) Annual Main Meeting in Seville (July 2019), respectively.

In the leaf, final size is determined by cell proliferation and cell expansion, which must be tightly coordinated in order to form a functional organ. Numerous leaf growth regulators have been identified, but their connections have only begun to be described. To illustrate these emerging connections that form the leaf growth regulatory network, Vercruysse *et al.* 2020 integrate our current knowledge of six important gene regulatory modules that are involved in cell proliferation in Arabidopsis. When mutated or overexpressed, numerous genes from these modules produce enlarged leaves, often with increased cell division. In hybrids, heterosis in leaf size is also often associated with an increased number of cells. Liu *et al.* 2020 show that, in *Arabidopsis thaliana* hybrids, photosynthetic efficiency per unit leaf area in Arabidopsis hybrids does not drive heterosis, but faster development and increased leaf area result in higher photosynthate production per plant, thus contributing to biomass heterosis.

While leaves are the primary organs for photosynthesis, and hence are pivotal for plant growth and development, roots are also essential since they permit proper soil anchorage, microbial interactions, and effective water and nutrient uptake. The root system architecture (RSA) describes the spatial distribution of a root system within the soil and it is determined by the growth of the different root sub-parts including primary, lateral, and adventitious roots. In their review, Deja-Muylle *et al* 2020. present different phenotyping strategies (root traits and approaches) used for RSA analysis and an overview of the progress being made to find fundamental RSA genetic pathways by applying genome-wide association studies. At the cellular level, roots present a longitudinal arrangement along an axis, where a meristematic zone, an elongation zone, and a differentiation zone can be distinguished. Salvi *et al* 2020. review recent findings regarding the regulatory networks involved in root zonation and, in particular, the mechanisms involved in maintaining the position of the transition zone during root growth. The growth of the different root sub-parts is influenced by external stimuli, such as nutrients and other environmental cues, as well as by developmentally programmed signals, such as phytohormones. These influences are reviewed by Waidmann *et al.* 2020 who describe the similarities and differences between primary and lateral root organ growth, in response to environmental cues and phytohormones. Vissenberg *et al.* 2020 also illustrate the impact of hormones and changes in rhizosphere properties on the development of root hairs, the single-cell, tubular extensions of the epidermis that increase the absorptive surface of the root as the result of the extreme polarized growth at their apex.

Plant reproduction depends on successful fertilization of ovules by sperm cells. To create fit and competitive offspring, some plants prevent inbreeding and self-fertilization by the mechanism of self-incompatibility (SI). Wang *et al.* 2020 exploit genetically encoded fluorescent probes in order to study SI- induced cellular alterations. They describe SI-induced signalling that leads to changes in cytoplasmic ion-homeostasis, actin, endocytosis, and vacuole morphology in incompatible pollen. Compatible pollen grains function in male sperm-cell delivery at the female gametophytes by producing pollen tubes that rapidly elongate, penetrate, and navigate through female tissues. Their growth is very responsive to external cues, which guide their polarized growth machinery. Guo and Yang 2020 summarize the contribution of Rho-like GTPases (ROPs) to this controlled cell-surface expansion at the pollen tube tip by exo- and endocytosis.

We hope that this special issue on plant organ and tip growth will help to stimulate increased research on this exciting and challenging topic.

## References

[CIT0003] Deja-Muylle A, Parizot B, Motte H, Beeckman T 2020 Exploiting natural variation in root system architecture via genome wide association studies. Journal of Experimental Botany 71, 2379–2389. 10.1093/jxb/eraa029 31957786

[CIT0006] Guo J, Yang Z 2020 Exocytosis and endocytosis: coordinating and fine-tuning polar tip growth domain in pollen tubes. Journal of Experimental Botany 71, 2428–2438. 10.1093/jxb/eraa134 PMC7178420 32173729

[CIT0007] Liu P-C, Peacock WJ, Wang L, Furbank RT, Larkum AW, Dennis ES 2020 Leaf growth in early development is key to biomass heterosis in Arabidopsis. Journal of Experimental Botany 71, 2439–2450. 10.1093/jxb/eraa006 PMC7178430 31960925

[CIT0005] Salvi E, Di Mambro R, Sabatini S 2020 Dissecting mechanisms in root growth from the transition zone perspective. Journal of Experimental Botany 71, 2390–2396. 10.1093/jxb/eraa079 32064533

[CIT0001] Vercruysse J, Baekelandt A, Gonzalez N, Inzé D 2020 Molecular networks regulating cell division during Arabidopsis leaf growth. Journal of Experimental Botany 71, 2365–2378. 10.1093/jxb/erz522 PMC7178401 31748815

[CIT0004] Vissenberg K, Claeijs N, Balcerowicz D, Schoenaers S 2020 Hormonal regulation of root hair growth and responses to the environment in Arabidopsis thaliana. Journal of Experimental Botany 71, 2412–2427. 10.1093/jxb/eraa048 PMC7178432 31993645

[CIT0002] Waidmann S, Sarkel E, Kleine-Vehn J 2020 Same same, but different: growth responses of primary and lateral roots. Journal of Experimental Botany 71, 2397–2411. 10.1093/jxb/eraa027 PMC7178446 31956903

[CIT0008] Wang L, Trivino M, Lin Z, Carli J, Eaves D, Van Damme D, Nowack MK, Franklin-Tong VE, Bosch M 2020 New opportunities and insights into *Papaver* self-incompatibility by imaging engineered Arabidopsis pollen. Journal of Experimental Botany 71, 2451–2463. 10.1093/jxb/eraa092 PMC7178406 32100005

